# The Protective Role of Ambient Ultraviolet Radiation Against Dementia: An Ecological Analysis of Global Data

**DOI:** 10.1002/hsr2.70302

**Published:** 2025-01-07

**Authors:** Wenpeng You

**Affiliations:** ^1^ Adelaide Medical School The University of Adelaide Adelaide South Australia Australia; ^2^ School of Nursing and Midwifery Western Sydney University Penrith New South Wales Australia; ^3^ Adelaide Nursing School The University of Adelaide Adelaide South Australia Australia

**Keywords:** ambient ultraviolet radiation, dementia prevention, ecological analysis, neuroinflammation, public health strategies, vitamin D synthesis

## Abstract

**Background and Aims:**

This study investigates the global impact of ambient ultraviolet radiation (UVR) on dementia incidence, addressing its controversial association with dementia risk. UVR, through both vitamin D‐dependent and independent mechanisms, influences physiological processes essential for brain health, such as reducing neuroinflammation, improving sleep regulation, and enhancing neuroplasticity. This study aims to clarify the relationship between UVR and dementia incidence and evaluate its role in public health strategies for dementia prevention.

**Methods:**

An ecological analysis across 204 countries was conducted using country‐specific data on UVR levels and dementia incidence rates. Bivariate analysis, partial correlation, and multiple linear regression models were employed to assess the relationship between UVR and dementia incidence. Confounding factors, including aging, economic affluence, genetic predisposition, and urbanization, were controlled to ensure robust results. Subgroup analyses were performed to explore differences across income classifications, regional groupings, and developmental statuses, based on World Bank and United Nations criteria.

**Results:**

A significant inverse correlation between UVR and dementia incidence was identified (*r* = −0.764, *p* < 0.001), which persisted after adjusting for confounding factors. UVR emerged as the strongest predictor of dementia incidence, explaining a substantial portion of the variance, followed by aging as the second strongest predictor. Subgroup analyses revealed that the protective effects of UVR were particularly pronounced in developing countries, where limited access to vitamin D supplementation, combined with greater exposure to natural sunlight may enhance its influence.

**Conclusion:**

Lower ambient UVR levels are associated with higher dementia incidence rates worldwide, suggesting a critical role for UVR in mitigating dementia risk. Public health strategies should consider environmental factors like UVR, particularly in regions with limited sunlight. Incorporating interventions to optimize UVR exposure could offer a cost‐effective approach to reducing the global dementia burden and improving brain health outcomes.

## 1 Background

Dementia, characterized by a progressive decline in cognitive abilities, significantly impacts daily functioning and encompasses conditions like Alzheimer's disease and vascular dementia. Affecting around 5% of people aged 65 and older, dementia ranks as a leading cause of disability and dependency worldwide, placing a substantial burden on families, healthcare systems, and economies [1, 2].

As populations age and no cure for dementia exists, focusing on modifiable risk factors has become a crucial public health priority. While age and genetics are primary, nonmodifiable risk factors [3], environmental influences have gained attention in recent years. Lifestyle factors like excessive alcohol consumption [4], smoking [5], and physical inactivity [6] have been linked to a higher risk of dementia. Comorbid conditions, including diabetes, obesity, and cardiovascular diseases, further increase this risk [7].

Among environmental factors, climate conditions, particularly temperature variations, have been linked to dementia onset [8, 9]. However, ambient ultraviolet radiation (UVR), which plays a crucial role in regulating the human body, brain, and immune system [10], is another significant environmental factor for maintaining cognitive health. Despite its relatively stable nature, UVR has received limited attention in dementia research. It may influence dementia development through its impact on key physiological processes that support brain health [11, 12].

Sunlight, the primary source of UVR, plays a vital role in producing vitamin D in the skin. Vitamin D has neuroprotective properties, supporting neuron growth and survival, and its deficiency has been linked to an increased risk of cognitive decline and dementia. Additionally, vitamin D modulates immune responses, reducing brain inflammation—a key factor in neurodegenerative diseases [13].

Sunlight also helps regulate circadian rhythms [14], improving sleep patterns essential for brain health. Proper sleep quality is linked to a lower risk of cognitive decline and dementia [15]. Daytime sunlight exposure enhances nighttime melatonin production, which regulates sleep‐wake cycles and protects brain cells from oxidative damage. Sunlight also promotes cardiovascular health by lowering blood pressure and reducing the risk of cardiovascular diseases, closely related to brain health [14, 16].

Sunlight positively impacts mental health by boosting mood and reducing the risk of depression, a known risk factor for dementia, through the elevation of serotonin levels [17, 18]. Regular exposure to natural light has been associated with improved cognitive function and slower cognitive decline in older adults [19]. Sunlight also supports neuroplasticity, crucial for maintaining cognitive health and preventing neurodegeneration, while its anti‐inflammatory properties provide additional protection to the brain [20]. Light therapy using natural or artificial light has been employed to manage dementia symptoms such as sleep disturbances and agitation [21]. Furthermore, recent studies have highlighted the potential roles of UVB‐generated lumisterol and tachysterol as additional prohormones [22, 23]. Similar to vitamin D, these compounds are activated by UVB radiation and contribute to various physiological processes, including skin health, cognitive function, and systemic effects [22, 23]. Together, these findings underscore the multifaceted benefits of sunlight and UVB radiation for brain health and overall well‐being.

Exploring sunlight's role in preventing cognitive decline and promoting brain health is essential. Although prospective studies provide valuable insights into the vitamin D‐cognition relationship, they often require long timeframes [24]. Additionally, earlier research may not fully capture the impact of low sunlight exposure on dementia risk. Epidemiological studies, using advanced statistical methods, offer a robust alternative. However, data on everyday sunlight exposure and dementia onset remains scarce.

To address this gap, the study examines UVR's predictive role in global and regional dementia incidence rates (DIRs). Using empirical data and considering factors like life expectancy, economic status, genetic predisposition, and urbanization, this research assesses UVR's unique contribution to dementia. By exploring the relationship between UVR and dementia, the study aims to inform public health strategies and policies to mitigate the global dementia burden.

## 2 Methods

### 2.1 Data Sources

In this study, the dependent variable is the DIR, sourced from the Institute for Health Metrics and Evaluation (IHME) [25]. DIR is expressed as the number of newly diagnosed dementia cases per 100,000 people in 2019.

The independent variable, average daily ambient UVR, measured in joules per square meter (J/m²), was sourced from the WHO Global Health Observatory (GHO) [26]. UVR spans the 280–400 nm spectrum, with UVB radiation (280–315 nm) being specifically responsible for cutaneous vitamin D synthesis. This process converts vitamin D, initially a biologically inactive prohormone, into its active form. The data used represent annual ambient erythemal weighted UVR, which accounts for biological effects on human skin by applying the Erythema Action Spectrum. These values are calculated using satellite data or proxies such as latitudinal position to reflect population‐level exposure. Low UVR levels indicate consistently low ambient UVR throughout the year. The WHO provides these data to evaluate the disease burden associated with UVR exposure and its health impacts [26].

Based on previous studies, four potential confounding variables were included to analyze the independent role of birth rate in predicting DIR:

1. Economic affluence, indexed with per capita GDP purchasing power rate (GDP PPP in 2018 international $), was chosen and downloaded from the World Bank data repository [27] because it is associated with dementia risk [28]. This variable takes into account the relative cost of local goods, services, and inflation rates of the country.
2. Aging, measured with life expectancy at birth (e(0)), which reflects the aging process at the population level, was downloaded from the World Bank data repository [29]. Although dementia can occur at any stage of life, it predominantly affects older individuals [30]. Therefore, life expectancy at birth in 2018 is used to index the aging process.
3. Dementia genetic predisposition, quantified by the Biological State Index (I_bs_), which gauges the extent of dementia gene predisposition in a population, was sourced from a 2018 publication [31]. It is hypothesized that reduced natural selection, as indicated by I_bs_, may have facilitated the accumulation of deleterious genes associated with noncommunicable diseases such as dementia [32]. The I_bs_ specifically measures the genetic predisposition to DIRs attributable to diminished natural selection.
4. Urbanization data, represented by the country‐specific percentage of the population living in urban areas in 2018 [33], was sourced from the World Bank data repository. Urbanization is considered a significant predictor of dementia because it reflects major demographic shifts that involve lifestyle changes and also indicates the level of healthcare access within a country [34].

Ethical approval was not required for this study because all data were obtained from publicly available sources, including the Institute for Health Metrics and Evaluation and the United Nations (UN) agencies' websites. A consent statement is not applicable since the research did not involve individual human participants or animals.

### 2.2 Data Selection

A comprehensive data set of DIRs for 204 countries was obtained from the IHME [25]. To analyze this data, additional variables—UVR, life expectancy at birth (aging), economic affluence, genetic predisposition, and urbanization—were matched individually with this list using country‐specific information from the UN and its agencies. During the analysis, each country was treated as a separate unit of study, though not all had data for every variable. It is important to clarify that in this context, the term “country” refers to a reporting unit used by various international organizations rather than a sovereign nation, with “location,” “population,” and “country” being interchangeable terms for a single data reporting unit.

### 2.3 Data Collinearity Check

Multicollinearity is a most common issue leading to low data quality for regression model analysis. The underlying reason is that independent and confounding variables show high intercorrelations, which can make regression model results less reliable. This issue was ruled out through statistically calculating the correlation between dependent variable (DIR) and each of the five variables (predicting and confounding variables) with the multiple regression linear enter regression model (tolerance ≥ 0.20 and VIF ≤ 5). The collinearity criteria were tolerance > 0.20 and VIF < 5 as per the set criteria [35]. The results were reported in Table 1–(1) while exploring correlation coefficient between each predicting variable and DIR.

**Table 1:** Multiple linear regression to identify the significant predictors of dementia incidence rate and to check collinearity statistics.

(1): Enter	Dependent variable: Dementia incidence rate All countries, *n* = 204		Dependent variable: Dementia incidence rate All countries, *n* = 204			Dependent variable: Dementia incidence rate All countries, *n* = 204		
Predictor	Beta	Significance	Predictor	Beta	Significance	Collinearity statistics		
						Predictor	Tolerance	VIF
UV radiation	Not applicable		UV radiation	−0.545	< 0.001	UV radiation	0.637	1.571
Genetic predisposition	−0.124	0.279	Genetic predisposition	−0.167	0.065	Genetic predisposition	0.211	4.735
Economic affluence	0.064	0.457	Economic affluence	−0.058	0.403	Economic affluence	0.359	2.785
Urbanization	0.041	0.563	Urbanization	−0.020	0.720	Urbanization	0.552	1.812
Aging	0.750	< 0.001	Aging	0.604	< 0.001	Aging	0.142	7.055

(2): Stepwise		Dependent variable: Dementia incidence rate All countries, *n* = 204			Dependent variable: Dementia incidence rate All countries, *n* = 204				
Rank	Predictor	Beta	Significance	Adjust *R* ^2^	Rank	Predictor	Beta	Significance	Adjust *R* ^2^
	UV radiation	Not incorporated			1	UV radiation	−0.532	< 0.001	0.581
1	Aging	0.712	< 0.001	0.504	2	Aging	0.410	< 0.001	0.694
2	Economic affluence	Not significant				Economic affluence	Not significant		
	Genetic predisposition	Not significant				Genetic predisposition	Not significant		
	Urbanization	Not significant				Urbanization	Not significant		

*Note:* Ultraviolet Radiation (UVR), expressed as the average daily ambient ultraviolet radiation level (in J/m^2^), the World Health Organization; Dementia incidence rate, the number of new cases per 100,000 people diagnosed in 2019 Institute for Health Metrics and Evaluation of University of Washington; Economic affluence, per capita GDP PPP, measured with the per capita purchasing power parity (PPP) value of all final goods and services produced within a territory in a given year (2018), the World Bank; Genetic predisposition (Biological State Index, I_bs_), dementia genetic background predisposition level due to reduced natural selection, downloaded from previous publication [32]; Aging, measured with life expectancy at birth (e(0)), the number of years a newborn infant would live if prevailing patterns of mortality at the time of its birth were to stay the same throughout its life, 2018, the World Bank.

### 2.4 Data Analysis

To assess the relationship between UVR and DIR at the population level, the analysis was conducted in five steps [36, 37, 38].

1. Scatter Plots: Visual analysis was performed using Microsoft Excel to create scatter plots with the original data. These plots helped assess data quality by showing the distribution and relationships within the data set.
2. Bivariate Correlations: Pearson's *r* and Spearman's rho were used to calculate bivariate correlations among six variables (UVR, DIR, aging, economic affluence, genetic predisposition, and urbanization). Pearson's *r* assumes normality and linearity, while Spearman's rho is a nonparametric measure of rank correlation. By using both methods, we can ensure a robust analysis that accounts for potential deviations from normality.
3. Multiple Linear Regression: Standard multiple linear regression (enter method) was used to describe the correlations between the dependent variable (DIR) and the predicting variables (UVR). We performed multiple linear regression with two models: one incorporating UVR as a predictor and one excluding UVR. Additionally, standard multiple linear regression (stepwise method) was used to select the predictor(s) with the greatest influence on DIR in two versions: one incorporating UVR and one excluding UVR.
4. Partial Correlations: To refine the analysis, partial correlations were computed. This measures the relationship between two variables while controlling for the influence of other variables. By incorporating partial correlation, we can better understand the direct associations between variables and exclude the effects of confounding factors. Each variable was alternated as the independent predictor, while the other variables were included as potential confounding factors.
5. Comparative Analysis: To gain a comprehensive understanding of the correlation between UVR and DIR across different global contexts, this study conducted analyses with grouped populations. These groupings allowed for a comparative analysis of the strength of the correlation between UVR and DIR among various country classifications. Countries were categorized based on the World Bank income classifications (low‐ and middle‐income and high‐income countries), UN common practice and WHO regional classifications, and other groups determined by geography, culture, development role, or socioeconomic status. Specific groupings included the Asia Cooperation Dialog, Asia‐Pacific Economic Cooperation, the Arab World, the European Economic Area, countries where English is the official language, Latin America, Latin America and the Caribbean, the Organization for Economic Co‐operation and Development, the Southern African Development Community, and the Shanghai Cooperation Organization. Each grouping was chosen to represent distinct characteristics that could influence health outcomes, i.e. DIR. The specific country included in each grouping was collected from the respective organizations' official websites, enabling a detailed analysis of how geographical and socioeconomic factors globally impact dementia incidence.
6. Fisher *r*‐to‐*z* Transformation: Given that the World Health Organization reports over 55 million people with dementia worldwide, with more than 60% residing in low‐ and middle‐income countries, a Fisher *r*‐to‐*z* transformation was conducted to compare the role of UVR in predicting DIR. The aim of this transformation was to compare the importance of UVR in determining dementia between low‐ and middle‐income countries and high‐income countries, as well as between UN‐developing and UN‐developed countries.

Bivariate correlations, partial correlation, and multiple linear regression (using both the enter and stepwise methods) were performed in the Statistical Package for the Social Sciences (SPSS) version 29. All tests were two‐sided. Statistical significance was reported at a *p* value of less than 0.05, with additional levels of significance reported for *p* < 0.01 and *p* < 0.001. The regression analysis criteria were set at a probability of F to enter less than or equal to 0.05 and to remove greater than or equal to 0.10. Scatter plots were created in Excel 2016 using the raw data.

## 3 Results

**Figure 1:**
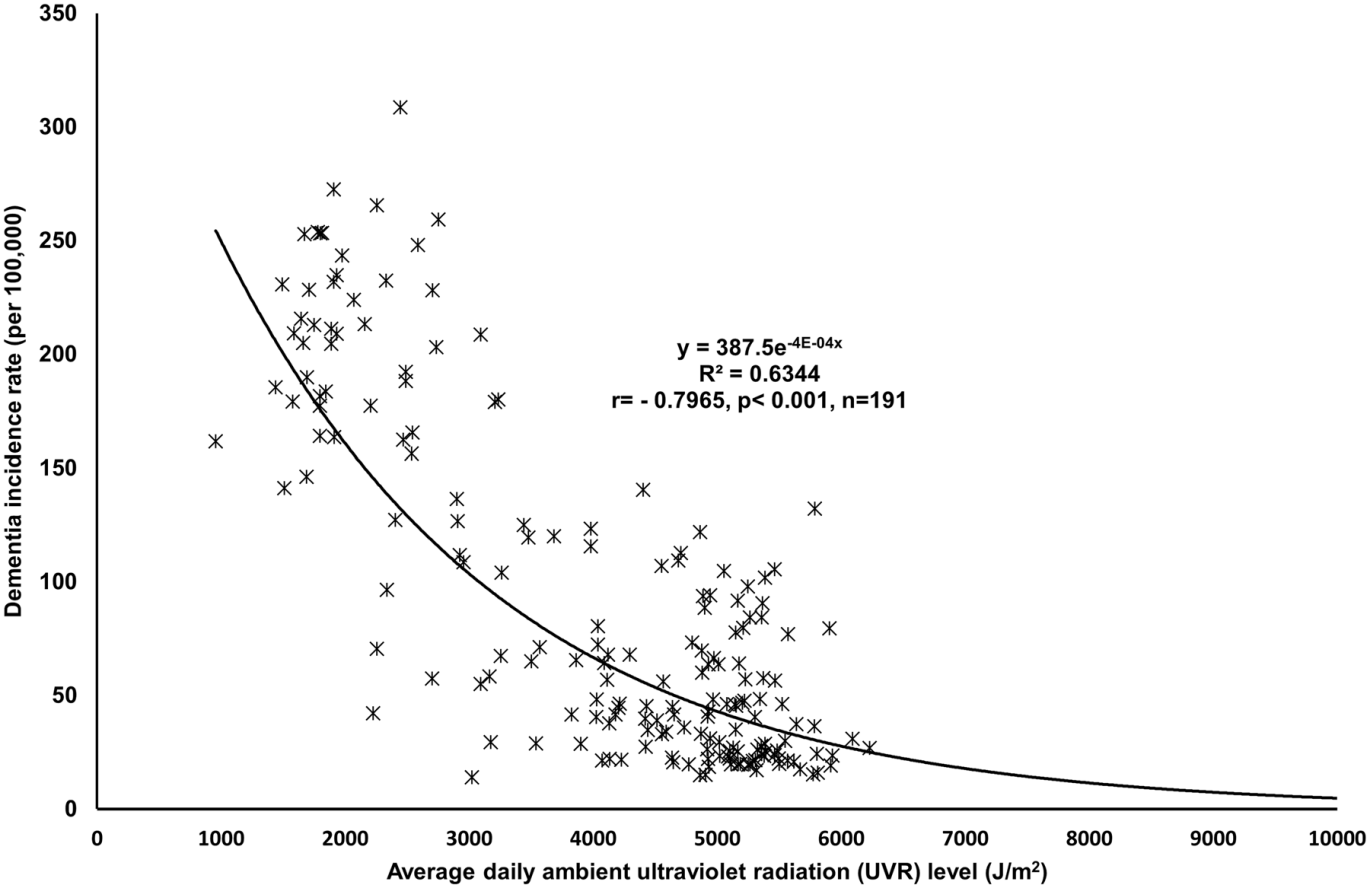
Plot to show the relationship between ultraviolet radiation and dementia incidence rate worldwide.

The scatterplots revealed a power relationship between UVR and DIR, with a strong and negative correlation. The *R* ^2^ value of 0.6344 (*r* = −0.7965, *p* < 0.001, *n* = 191, Figure 1) quantifies this. Overall, there is a significant trend showing that countries with higher UVR tend to have lower DIRs globally.

The scatterplots confirmed a strong and inverse relationship between UVR and DIR, which was further confirmed through standard multiple linear regression analyses, which were conducted to predict DIR, considering UVR, aging, economic affluence, genetic predisposition, and urbanization as the predicting variables.

Both Pearson's *r* and nonparametric models revealed a significant negative correlation between UVR and DIR on a global scale (*r* = −0.764, rho = −0.704, *p* < 0.001) (Table 2). Additionally, aging, economic affluence, genetic predisposition, and urbanization were found to have moderate to strong correlations with DIR, as indicated by both Pearson and nonparametric analyses (Table 2). However, in multiple linear regression and partial correlation analyses, economic affluence, genetic predisposition, and urbanization did not show significant correlations, suggesting that their predictive roles may be highly confounded.

**Table 2:** Pearson's *r* and nonparametric correlation matrix between all variables.

	UV radiation	Dementia incidence	Genetic predisposition	Economic affluence	Urbanization	Aging
UV radiation	1	−0.764***	−0.482***	−0.549***	−0.464***	−0.565***
Dementia Incidence	−0.704***	1	0.590***	0.582***	0.483***	0.717***
Genetic predisposition	−0.630***	0.833***	1	0.567***	0.523***	0.876***
Affluence	−0.624***	0.759***	0.895***	1	0.649***	0.733***
Urbanization	−0.473***	0.510***	0.630***	0.720***	1	0.604***
Aging	−0.610***	0.810***	0.930***	0.880***	0.640***	1

*Note:* Pearson *r* (above diagonal) and nonparametric rho (below diagonal) correlations were reported. Ultraviolet Radiation (UVR), expressed as the average daily ambient ultraviolet radiation level (in J/m^2^), the World Health Organization; Dementia incidence rate, the number of new cases per 100,000 people diagnosed in 2019 Institute for Health Metrics and Evaluation of University of Washington; Economic affluence, per capita GDP PPP, measured with the per capita purchasing power parity (PPP) value of all final goods and services produced within a territory in a given year (2018), the World Bank; Genetic predisposition (Biological State Index, I_bs_), dementia genetic background predisposition level due to reduced natural selection, downloaded from previous publication [32]; Aging, measured with life expectancy at birth (e(0)), the number of years a newborn infant would live if prevailing patterns of mortality at the time of its birth were to stay the same throughout its life, 2018, the World Bank.

*** Significance levels: *p* < 0.001. Number of country range, 181–198.

Multiple linear regression models were performed to identify the statistical role of each of the five variables in predicting DIR, and accordingly, their roles were ranked. When UVR was excluded as a predicting variable, only aging showed a significant correlation with DIR (Beta = 0.750, *p* < 0.001). However, when UVR was included as a predictor, both UVR and aging showed significant correlations with DIR (Beta = −0.545 and 0.604, respectively, *p* < 0.001). Each of the other three predictors (genetic predisposition, economic affluence, and urbanization) showed negligible and insignificant correlations with DIR (Table 1–(1)).

Similarly, in a stepwise linear regression model, when UVR was not included as a predictor, aging was identified as the only variable significantly influencing the development of DIR (*R* ^2^ 0.504). However, when UVR was included along with the other five independent variables, UVR emerged as the most influential predictor of DIR with an *R* ^2^ of 0.581, followed by aging, increasing *R* ^2^ to 0.694. Genetic predisposition, economic affluence, and urbanization did not show significant influence on DIR, and therefore they were not selected as the significant variables contributing to DIR. This suggests that genetic predisposition, economic affluence, and urbanization did not account for a major part of the impact on DIR. These findings support the previous suggestion that UVR and aging were significant predictors of DIR in the partial correlation analyses and scatter plots.

The relationship between DIR and each potential confounding variable and predicting variable (UVR, aging, economic affluence, genetic predisposition, and urbanization) was examined using partial correlation analysis. In this analysis, the other four variables were statistically adjusted to explore the independent correlation between DIR and the fifth variable. The results indicated a strong and significant correlation between DIR and UVR (*r* = −0.621, *p* < 0.001) regardless of the other four variables (Table 3–(1)). Aging also showed a significant correlation with DIR, although the correlation coefficient was weak to moderate (*r* = 0.384, *p* < 0.001). On the other hand, economic affluence, genetic predisposition, and urbanization had minimal correlations with DIR, suggesting that they did not independently correlate with DIR (Table 3–(1)).

**Table 3:** Comparative partial correlation coefficients for dementia incidence rate and predicting variables across various combinations of controlled variables.

Variables	(1): UV Radiation, economic affluence, genetic predisposition, aging, and urbanization were alternated as the predicting variable for calculating its independent relationship with dementia incidence, while the other four variables were statistically kept constant.														
	Dementia incidence			Dementia incidence			Dementia incidence			Dementia incidence			Dementia incidence		
	*r*	*p*	*df*	*R*	*p*	*df*	*r*	*p*	*df*	*r*	*p*	*df*	*r*	*p*	*df*
UV radiation	−0.621	< 0.001	175	—	—	—	—	—	—	—	—	—	—	—	—
Economic affluence	—	—	—	0.061	0.422	175	—	—	—	—	—	—	—	—	—
Genetic predisposition	—	—	—	—	—	—	−0.127	0.920	175	—	—	—	—	—	—
Aging	—	—	—	—	—	—	—	—	—	0.384	< 0.001	175	—	—	—
Urbanization	—	—	—	—	—	—	—	—	—	—	—	—	−0.011	0.889	175

Variables	(2): UV Radiation, genetic predisposition, aging, and urbanization were alternated as the individual potential confounder for exploring the independent correlations between dementia incidence and the other four independent variables.														
	Dementia incidence			Dementia incidence			Dementia incidence			Dementia incidence			Dementia incidence		
	*r*	*p*	*df*	*r*	*p*	*df*	*r*	*p*	*df*	*r*	*p*	*df*	*r*	*p*	*df*
UV Radiation	—	—	—	−0.624	< 0.001	182	−0.655	< 0.001	178	−0.679	< 0.001	185	−0.697	< 0.001	186
Aging	0.535	< 0.001	182	—	—	—	0.524	< 0.001	180	0.513	< 0.001	183	0.609	< 0.001	189
Economic affluence	0.301	< 0.001	178	0.119	0.109	180	—	—	—	0.372	< 0.001	178	0.403	< 0.001	182
Genetic predisposition	0.393	< 0.001	185	−0.111	0.132	183	0.388	< 0.001	178	—	—	—	0.452	< 0.001	185
Urbanization	0.225	< 0.010	186	0.091	0.212	189	0.170	< 0.050	182	0.253	< 0.001	185	—	—	—

*Note:* Ultraviolet Radiation (UVR), expressed as the average daily ambient ultraviolet radiation level (in J/m^2^), the World Health Organization; Dementia incidence rate, the number of new cases per 100,000 people diagnosed in 2019 Institute for Health Metrics and Evaluation of University of Washington; Economic affluence, per capita GDP PPP, measured with the per capita purchasing power parity (PPP) value of all final goods and services produced within a territory in a given year (2018), the World Bank; Genetic predisposition (Biological State Index, I_bs_), dementia genetic background predisposition level due to reduced natural selection, downloaded from previous publication [32]; Aging, measured with life expectancy at birth (e(0)), the number of years a newborn infant would live if prevailing patterns of mortality at the time of its birth were to stay the same throughout its life, 2018, the World Bank.

When each of the five variables (UVR, aging, economic affluence, genetic predisposition, and urbanization) was kept constant, the partial correlation coefficient between DIR and the other four variables was explored. UVR and aging were the only two variables that correlated with DIR independent of the other four variables individually (Table 3–(2)). Interestingly, UVR significantly correlated with DIR independent of aging, economic affluence, genetic predisposition, and urbanization individually and in combination (Table 3–(2)), with very similar coefficients (−0.621 to −0.697). This suggests that the unique contribution of low UVR to DIR cannot be explained by the four variables individually or in combination.

Table 4 presents the correlation between UVR and DIR for different country groupings. The strength and significance of the correlations varied based on sample size and country characteristics. In UN‐developing countries, UVR had a significantly stronger correlation with DIR compared to UN‐developed countries in both Pearson's *r* and nonparametric models (*z* = −3.87 and −3.51, respectively, *p* < 0.001). Conversely, the correlation coefficients between World Bank LMICs and high‐income countries were negligible in both Pearson's *r* and nonparametric models, as indicated by small *z*‐scores and *p* values over 0.050. When countries with geographic proximities were grouped together, the correlation coefficients were generally very weak and/or insignificant, as observed in WHO Africa, WHO South‐East Asia, the European Economic Area, and the Southern African Development Community (Table 4). However, UVR showed moderate to strong correlation coefficients with DIR in all other country groupings (Table 4).

**Table 4:** Ultraviolet Radiation determining dementia incidence rate in different country groupings.

Country groupings	Pearson *r*	*p*	Nonparametric	*p*	*n*
Worldwide	−0.764	<0.001	−0.704	< 0.001	191
World Bank income classifications					
High income	−0.733	< 0.001	−0.654	< 0.001	63
Upper middle income	−0.596	< 0.001	−0.519	< 0.001	51
Low middle income	−0.610	< 0.001	−0.384	< 0.010	49
Low income	−0.479	< 0.010	−0.312	0.106	28
Low‐ and middle‐income countries (LMIC)	−0.620	< 0.001	−.524	< 0.001	128
Fisher *r*‐to‐*z* transformation (LMIC vs. High‐income)	*z* = 1.34, *p* = 0.090		*z* = 1.28, *p* = 0.100		
United Nations (common practice)					
Developed	0.200	0.193	0.190	0.217	44
Developing	−0.448	< 0.001	−0.405	< 0.001	147
Fisher *r*‐to‐*z* transformation (Developing vs. developed)	*z* = −3.87, *p* < 0.001		*z* = −3.51, *p* < 0.001		
WHO regions					
Africa	−0.289	0.052	−0.056	< 0.001	47
Americas	−0.682	< 0.001	−0.224	0.197	35
Eastern Mediterranean	−0.748	< 0.001	−0.738	< 0.001	21
Europe	−0.504	< 0.001	−0.322	< 0.050	51
South‐East Asia	−0.281	0.403	−0.209	0.537	11
Western Pacific	−0.575	0.002	−0.446	0.020	27
Countries grouped with various factors					
Asia Cooperation Dialog	−0.459	< 0.050	−0.392	< 0.050	30
Asia‐Pacific Economic Cooperation	−0.739	< 0.001	−0.868	< 0.001	19
Arab World	−0.798	< 0.001	−0.748	< 0.001	21
European Economic Area	0.146	0.449	0.398	< 0.033	29
English as Official Language	−0.756	< 0.001	−0.474	< 0.001	56
Latin America	−0.633	< 0.001	−0.400	0.058	23
Latin America and Caribbean	−0.388	< 0.050	−0.074	0.684	33
Organization for Economic Co‐operation and Development	−0.416	< 0.010	−0.141	0.406	37
Southern African Development Community	0.120	0.658	0.129	0.633	16
Shanghai Cooperation Organization	−0.484	0.012	−0.561	0.003	26

*Note:* Ultraviolet Radiation (UVR), expressed as the average daily ambient ultraviolet radiation level (in J/m^2^), the World Health Organization; Dementia incidence rate (DIR), the number of new cases per 100,000 people diagnosed in 2019 Institute for Health Metrics and Evaluation of the University of Washington.

## 4 Discussion

This study reveals a significant inverse relationship between UVR and DIRs globally. Higher UVR levels are associated with lower DIR, as evidenced by a strong negative correlation (*r* = −0.7965, *p* < 0.001), with an *R* ^2^ value of 0.6344. This suggests that UVR may offer protective benefits against dementia. Even after accounting for confounding factors like aging, affluence, genetic predisposition, and urbanization, UVR remains a prominent protective factor.

Research has increasingly recognized the potential link between UVR exposure and reduced dementia risk. UVR primarily promotes vitamin D synthesis [39], essential for cognitive health, and stimulates nitric oxide release [40], which enhances cerebral blood flow. Sunlight exposure during the day also plays a critical role in regulating circadian rhythms, indirectly supporting the synthesis and release of melatonin at night. Proper alignment of circadian rhythms improves sleep quality, an important factor for cognitive health and dementia prevention [41]. Although melatonin production itself is not directly linked to UVR and occurs primarily in response to the absence of light, the regulation of circadian rhythms through sunlight exposure contributes to overall cognitive function. Additionally, sunlight's mood‐enhancing effects may reduce dementia risk by alleviating depression, a known risk factor [42]. Together, these mechanisms highlight the multifaceted role of sunlight in promoting brain health and mitigating dementia risk.

Various studies support these findings through diverse research methods. For instance, cross‐sectional studies have examined the correlation between vitamin D levels and cognitive function in older adults, revealing that low vitamin D levels are associated with cognitive decline [43, 44]. Systematic reviews and meta‐analyses, such as those by Annweiler et al. [45], further reinforce the connection between vitamin D, sunlight exposure, and cognitive health.

Longitudinal studies, like those by Fratiglioni et al. [46], have tracked older adults over time to assess the impact of lifestyle factors on dementia risk, revealing the causal relationship between sunlight exposure and cognitive health. Experimental and clinical studies, such as those by Mahfoz et al. [47], explored the neuroprotective role of vitamin D using animal models to understand its biological mechanisms. Observational studies, including those by Ju et al. [48] and Videnovic et al. [49], examined the impact of sleep and circadian rhythms on neurodegenerative diseases, highlighting the protective role of light exposure [50].

These studies collectively demonstrate how UVR, and vitamin D synthesis contribute to brain health, potentially reducing dementia risk. Sunlight exposure supports neuroprotective mechanisms, reduces neuroinflammation, and improves cognitive functions. However, low UVR levels can lead to insufficient vitamin D synthesis, impacting temperature regulation and potentially increasing dementia risk.

Recent advances highlight the homeostatic role of UVR in protecting against dementia initiation through both vitamin D‐dependent and vitamin D‐independent mechanisms [10, 51], with the latter mediated by the activation of the skin's neuroendocrine system [52]. This system integrates sensory, hormonal, and immune signals, enabling the skin to function as a peripheral neuroendocrine organ that communicates with the central nervous system and the immune system [52]. By regulating inflammation, oxidative stress, and neuroprotection, these vitamin D‐independent pathways complement the well‐established vitamin D‐dependent effects [10, 51], providing a comprehensive physiological basis for the observed protective effects of UVR. This dual mechanism aligns with the findings in this study, highlighting the importance of both pathways in mitigating dementia risk and offering potential therapeutic targets for neurodegenerative diseases [10, 51].

Beyond vitamin D, UVB radiation also generates lumisterol and tachysterol, which function as prohormones and can be further activated. These compounds exhibit significant biological activity, as highlighted in recent research [22, 23]. Their roles in neuroprotection and other systemic effects could provide additional pathways through which UVR impacts health outcomes, including dementia risk. This expands the understanding of UVB's multifaceted effects and underscores the need for further exploration into these pathways [22, 23].

Despite these promising findings, the relationship between UVR and cognitive health is complex. While moderate UV exposure may offer cognitive benefits, excessive exposure poses risks, including skin damage and increased skin cancer risk [53, 54]. High UVR levels can also cause oxidative stress and inflammation, potentially damaging brain cells and contributing to neurodegeneration [53]. Environmental factors like air pollution, often associated with high UV exposure, have also been linked to negative effects on the nervous system and an increased risk of Alzheimer's disease [53].

The study also confirms aging as a significant predictor of DIR. Aging shows a strong positive correlation with DIR, reinforcing that age is a major risk factor for dementia. However, factors like economic affluence, genetic predisposition, and urbanization did not consistently correlate with DIR, suggesting that their impact is minimal compared to UVR and aging.

The weaker correlation between UVR and DIR in developed countries may reflect lifestyle differences, healthcare access, and lower baseline UV exposure levels. In contrast, developing countries showed a stronger negative correlation, possibly due to increased time spent outdoors leading to higher UVR exposure [44]. In many developing countries, occupational and cultural practices involve more outdoor activities, which can enhance UVR exposure and consequently amplify its protective effects against dementia through mechanisms like vitamin D synthesis and activation of the skin's neuroendocrine system [44]. Additionally, limited access to healthcare services and vitamin D supplementation in these regions makes natural sunlight a more critical source of vitamin D and other UVR‐mediated benefits [55]. Conversely, in developed countries, factors such as indoor lifestyles, higher urbanization rates, and prevalent use of sun protection measures may reduce actual UVR exposure despite high ambient UVR levels, potentially attenuating its protective effects on dementia risk [56]. This suggests that the amount of time spent outdoors may significantly influence the impact of UVR on cognitive health, and highlights the need for further individual‐level research to quantify outdoor exposure and its relationship with dementia incidence.

This study underscores the need for further research to balance the benefits of moderate UV exposure with potential risks, taking into account individual health conditions and environmental factors. The findings highlight the protective role of UVR against dementia, especially in developing regions, offering valuable insights for public health strategies and dementia prevention efforts.

### 4.1 Conclusion

Ambient UVR may significantly protect against dementia incidence worldwide. While aging emerged as a key risk factor, the minimal impact of economic affluence, genetic predisposition, and urbanization suggests that environmental factors like UV exposure could play a more prominent role in dementia prevention. These findings underscore the need to prioritize environmental and lifestyle factors in dementia research and prevention strategies.

### 4.2 Study Strength and Limitation

This study benefits from its use of ecological data, allowing for an examination of UVR's relationship with DIRs across countries. This broader scope is advantageous due to the low incidence of dementia, enabling the analysis of larger sample sizes compared to individual‐based studies and enhancing the ability to detect risk factors like UVR. However, limitations arise from the reliance on aggregated country‐level data, which restricts the identification of individual‐level risk factors and may obscure significant regional variations in UVR's impact. Additionally, the use of international data sources introduces potential errors and inconsistencies in data collection methods, and variations in dementia diagnosis accuracy due to administrative errors and reporting biases, especially in developing countries, can affect DIR calculations.

While the study provides strong evidence for an inverse relationship between UVR and DIR, its design limits causal inferences, and aggregate data may obscure individual‐level differences. Future research should investigate the mechanistic pathways connecting UVR to reduced dementia risk, focusing on vitamin D synthesis, inflammation, and neuroprotection. Longitudinal studies at the individual level could offer clearer insights into causality and better understand how UVR influences different types of dementia.

### 4.3 Implications for Public Health

Understanding the link between ambient UVR and dementia risk is essential for developing effective public health strategies. This research highlights the importance of considering environmental factors, particularly UVR, in efforts to reduce the global dementia burden. Addressing the effects of lower UVR exposure on vulnerable populations, particularly in regions with limited sunlight availability, could help mitigate dementia risk.

To translate these findings into practice, several targeted interventions are recommended. Encouraging outdoor activities and social engagement among older adults can safely increase UVR exposure while enhancing physical and mental well‐being. Enhancing building designs with features such as larger windows, skylights, and open spaces can improve access to natural light indoors, benefiting individuals who spend significant time inside, such as aged care residents. Urban planning initiatives that prioritize accessible parks and recreational areas can further support outdoor engagement and increased sunlight exposure.

Additionally, public health campaigns can educate populations on safe sun exposure practices, balancing the benefits of UVR with the risks of skin damage. In regions with limited natural sunlight, light therapy could replicate the benefits of UVR under controlled conditions. These sunlight‐sensitive strategies, informed by the study's findings of a negative correlation between UVR and dementia incidence, can provide actionable guidance for reducing dementia risk and improving public health outcomes globally.

## Author Contributions

Wenpeng You, as the sole author for this study, reviewed the literature and obtained the data for structuring this study. Wenpeng You formulated the hypothesis relating nurse‐midwifery density to neonatal mortality rate, analyzed the data and interpreted results and wrote the text. Wenpeng You approved the final version of the manuscript. All authors have read and approved the final version of the manuscript. Wenpeng You had full access to all of the data in this study and takes complete responsibility for the integrity of the data and the accuracy of the data analysis.

## Conflicts of Interest

The author declares no conflicts of interest.

### 1 Transparency Statement

The sole author, Wenpeng You affirms that this manuscript is an honest, accurate, and transparent account of the study being reported; that no important aspects of the study have been omitted; and that no discrepancies from the study has occurred.

## Supporting information

Supplementary information.

## Data Availability

All the data included in our data analyses are freely available from the United Nations agencies' online repositories which are open to the public. The data sources have been described in the section of “Materials and Methods.” There is no need to obtain formal permission to use the data for noncommercial purposes, which is compliant with the agency's public permission in their terms and conditions.
